# Development and Validation of Molecularly Imprinted Polymers with Bio-Based Monomers to Adsorb Carbamazepine from Wastewater

**DOI:** 10.3390/molecules30122533

**Published:** 2025-06-10

**Authors:** Elettra Savigni, Elisa Girometti, Laura Sisti, Frank Benstoem, Davide Pinelli, Dario Frascari

**Affiliations:** 1 Department of Civil, Chemical, Environmental and Materials Engineering, University of Bologna, Via Terracini 28, 40131 Bologna, Italy; elettra.savigni2@unibo.it (E.S.); elisa.girometti2@unibo.it (E.G.); davide.pinelli@unibo.it (D.P.); dario.frascari@unibo.it (D.F.); 2 ATD GmbH, Krefelder Straße 147, D-52070 Aachen, Germany; benstoem@atdgmbh.de

**Keywords:** pharmaceuticals, adsorption, MIP, bio-based monomers, wastewater treatment, emerging water contaminants

## Abstract

The removal of pharmaceutical contaminants like the anticonvulsant carbamazepine (CBZ) from water sources is a growing environmental challenge. This study explores the development of molecularly imprinted polymers (MIPs) tailored for CBZ adsorption using a bulk polymerization approach. Initially, this study focused on selecting the optimal cross-linker, comparing a trifunctional (trimethylolpropane triacrylate, TRIM) and a bifunctional cross-linker (ethylene glycol dimethacrylate, EGDMA) in combination with two common monomers (2-vinylpyridine and methacrylic acid). TRIM-based MIPs demonstrated superior adsorption efficiency and stability due to their higher cross-linking density. To improve sustainability, six bio-based monomers were investigated; of these, eugenol (EUG) and coumaric acid (COU) showed the best CBZ affinity due to π-π interactions and hydrogen bonding. Adsorption tests conducted in pharmaceutical-spiked real wastewater demonstrated that MIPs exhibit a high selectivity for CBZ over other pharmaceuticals like the anti-inflammatory drugs diclofenac (DCF) and ibuprofen (IBU), even at high concentrations. Reaction conditions were further optimized by adjusting the reaction time and the ratio between reagents to enhance selectivity and adsorption performance. These results highlight the potential of bio-based MIPs as efficient and selective materials for the removal of pharmaceutical pollutants from wastewater.

## 1. Introduction

Contaminants of emerging concern (CECs) are natural or synthetic chemicals increasingly detected in water bodies but not consistently monitored in the environment [1]. Recently, growing attention has been given to CECs due to their potential risks to ecosystems and human health. According to the United States Environmental Protection Agency (US EPA), CECs are newly identified chemicals with effects that remain poorly understood [2]. These contaminants, believed to pose significant risks, originate from various sources and are found in concentrations ranging from micrograms to nanograms per liter [1].

The list of CECs, including pharmaceuticals, personal care products, plasticizers, pesticides, industrial chemicals, and complexing agents, is extensive and continually expanding due to the introduction of new chemicals and changes in their use and disposal [1,3]. Pharmaceuticals are among the most critical environmental issues in industrialized countries due to their widespread use for treating human and veterinary ailments [1]. Commonly detected pharmaceuticals in drinking water and wastewater include fluoxetine, lipid-lowering drugs, anti-acids, ciprofloxacin, diclofenac, steroids, beta-blockers, and analgesics [4].

Wilkinson et al. [5] identified active pharmaceutical ingredients in rivers across more than 50% of the world’s countries, with carbamazepine (CBZ) being the most frequently detected among the 61 compounds analyzed. CBZ, used to treat trigeminal neuralgia and bipolar disorders, is highly persistent in water due to its stability and resistance to biodegradation [6]. Conventional wastewater treatment plants (WWTPs) are largely ineffective at removing pharmaceuticals, with only about 10% of CBZ being eliminated, resulting in considerable concentrations in effluents and surface waters [1,6]. For this reason, the Revised Urban Wastewater Directive 2024/3019 of the European Union imposed for WWTPs serving > 150,000 people equivalent a quaternary treatment aimed at the removal of 13 micropollutants, including CBZ [7].

Various removal technologies, such as advanced oxidation processes and nanofiltration, have been proposed for micropollutants like CBZ. However, these often require high investment and maintenance costs and may lead to secondary pollution from oxidation by-products [8]. One promising approach involves using an adsorption step after the water tertiary treatment [9]. Commonly used adsorbents include commercial resins or activated carbons, which exhibit very high adsorption capacity but feature a complex and costly desorption process. In addition, the development of materials featuring a high selectivity for pharmaceuticals is of interest in specific applications, such as real-time sensors [1,6,9].

In recent years, researchers have explored the potential of molecularly imprinted polymers (MIPs) as an alternative and more selective adsorbent for pharmaceutical removal. MIPs are synthetic polymers designed to have specific recognition sites for target molecules, enabling selective adsorption. While many studies focus on MIPs for non-steroidal anti-inflammatory drugs (NSAIDs), only a few address CBZ as a target. The adsorption capacity of these materials is due to a combination of steric effects and interactions with the polymer. MIPs could work with three types of interactions: covalent bond, semi-covalent bond, and non-covalent bond [10,11]. The first two interaction approaches require more drastic conditions for both preparation and desorption, as the formation and breaking of stronger bonds are necessary [11,12]. Most research, conducted in recent decades, has preferred to focus on an imprinting approach that exploits non-covalent bonds, thus allowing for the adsorption and desorption of pollutants based on the greater or lesser affinity between the polymer and the solvent used for washing. The non-covalent interactions used in molecularly imprinted technology are H-bond, ionic interactions, acid–basic interactions, and aromatic interactions like π-π stacking.

**Figure 1:**
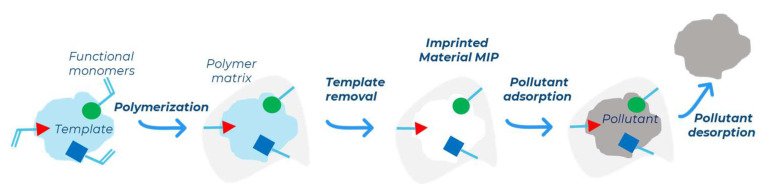
Schematic process to produce a molecularly imprinted material and recognition step.

Non-covalent MIPs are synthesized through a process called molecular imprinting, which involves radical polymerization. During this process, monomers surround the target molecules (template) and are then fixed using a cross-linker to set the disposition. The target molecule is subsequently removed by a washing step, leaving behind a polymer with cavities that are complementary in size, shape, and functional groups to the target compound. These polymers can selectively retain target analytes from complex samples. A schematic representation of molecular imprinting is depicted in Figure 1.

The shape of the cavities, the nature of the monomer, and the choice of porogen determine the nature and strength of the interactions between the target compounds and the imprinted polymer during extraction. Their stability under different pH, solvent, or temperature conditions makes MIPs excellent sorbents [13].

The main classical polymerization methods reported in the literature include bulk, precipitation, emulsion, and suspension polymerizations [10,11,12,14].

The bulk method involves only a small amount of solvent, resulting in a monolithic compound that must be grinded into powder after polymerization. The precipitation method, in contrast, requires a larger amount of solvent, leading to the formation of finer particles with respect to the bulk method. This approach eliminates the need for a grin-ding step, ensuring that all bonding sites created during the polymerization remain untouched.

Emulsion polymerization takes place within small droplets of an organic phase, dispersed in an aqueous phase, and stabilized by surfactants. Finally, suspension polymerization, a heterogeneous polymerization, is based on the suspension of droplets of a pre-polymerization mixture in a continuous phase (water, mineral oil, or perfluorocarbon). Each droplet acts like a mini bulk reactor, allowing for the production of spherical beads with a broad size range, from a few micrometers to millimeters.

Recent studies have focused on producing MIPs using green chemistry principles to develop more sustainable materials [15]. One example of these efforts is the testing of novel cross-linkers, including plant-based alternatives such as epoxidized soybean oil acrylate [16]. Other approaches include the use of alternative solvents, such as ionic liquids or deep eutectic solvents, as well as modifying synthesis techniques by employing ultrasound or microwave-assisted methods [17].

**Figure 2:**
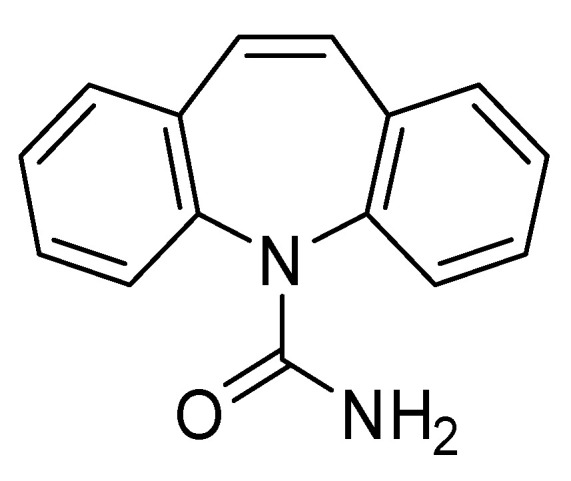
Carbamazepine.

**Figure 3:**
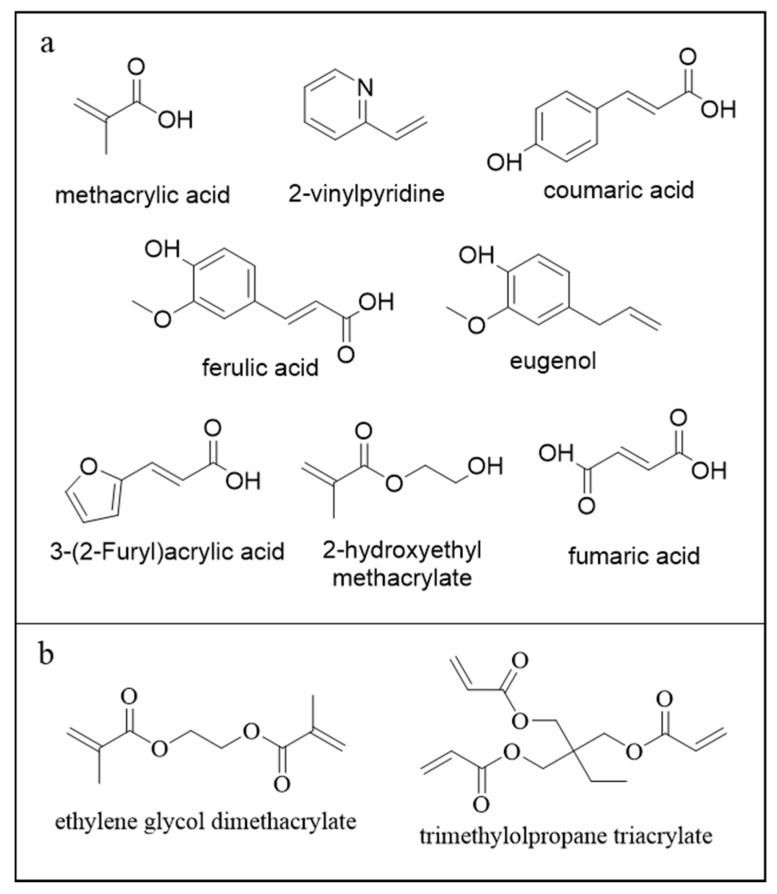
(**a**) Monomers; (**b**) cross-linkers.

This study aims to synthesize and characterize a MIP for the selective adsorption of CBZ (Figure 2) and to assess its adsorption performance. Common monomers used to adsorb CBZ are methacrylic acid (MAA) and 2-vinylpyridine (2VP) (Figure 3a) [13,18,19,20]. This study seeks to replace these monomers, in particular toxic 2VP, with bio-based alternatives. The literature presents various hypotheses on replacing conventional monomers with bio-based alternatives, such as polysaccharides or biomolecules like peptides [21]. In this study, compounds commonly used as templates have been re-evaluated and employed as functional monomers [22]. To develop novel materials, six monomers with different types of interactions were selected. Fumaric acid (FUM) was chosen to assess whether the presence of two acid groups improves adsorption compared to MAA. Eugenol (EUG) was selected as a possible substitute for 2VP, given its aromatic ring. Additionally, three monomers, with both acidic and aromatic functionalities, were studied: coumaric acid (COU) and ferulic acid (FER), both containing an aromatic ring, and 3-(2-furyl) acrylic acid (FAA), which features a furan ring. Finally, 2-hydroxyethyl methacrylate (HEMA), a monomer with an ester functionality, was investigated to compare a different functional group from those commonly reported in the literature (Figure 3a).

The cross-linking agent was chosen from either a difunctional or trifunctional option (ethylene glycol dimethacrylate (EGDMA) or trimethylolpropane triacrylate (TRIM)) based on the adsorption capacity of a MIP synthesized with 2VP as the monomer. The synthesis method adopted, although less technologically elaborate than microwave-assisted or ionic liquid-based approaches, distinguishes itself for its simplicity, cost-effectiveness, reproducibility, and, most importantly, scalability, making it a promising strategy for future applications beyond the laboratory scale, like in a quaternary step in a WWTP.

All synthesized materials were fully characterized using different techniques: FTIR, thermal analysis, and adsorption analysis. These analyses provide insight into the reaction status, polymer stability, and retention capacity for CBZ. For polymers prepared to optimize the reaction conditions, a reference material for each sample was also synthetized and compared with the corresponding MIP to verify the effectiveness of the imprinting.

This work presents two main novelties: (i) the development and optimization of MIPs for the adsorption of CBZ, an ubiquitous drug poorly investigated compared to NSAIDs such as ibuprofen, naproxen, diclofenac, or ketoprofen; (ii) no previous research has focused on the study of selective bio-based monomers targeting a pharmaceutical; instead, other studies have focused on the implementation of bio-based cross-linkers for the adsorption of micropollutants [16].

## 2. Results

In this study, the preparation of molecularly imprinted polymer (MIP) materials for the capture of carbamazepine (CBZ) was addressed using a readily scalable bulk polymerization technology adapted from the one reported by Cantarella et al. [23]. A pre-polymerization step was added, consisting of pre-mixing the monomer, the template, and the porogen for 20 min prior to the addition of the other polymerization components to promote the formation of the complex between the monomer and the target molecule [14]. Regarding the porogen, among the most frequently used solvents in similar studies (chloroform, DMSO, toluene, and acetonitrile), we chose acetonitrile due to its lower toxicity, in accordance with green chemistry principles.

A preliminary study was conducted to evaluate which cross-linker provides the best adsorption performance. Subsequently, different bio-based monomers were tested with the selected cross-linker. The monomers used in the screening were chosen to represent different functional groups, enabling the investigation of a range of potential interactions with the template molecule. Finally, to assess whether a longer reaction time affects the adsorption capacity and morphology, several MIPs were prepared using different reaction times. Additionally, a different monomer-to-cross-linker ratio was also tested.

Details about the molar composition, reagents employed, reaction time, and polymerization temperature of all syntheses, together with sample codes, are reported in Table 1. Acronyms are displayed in the acronym list.

### 2.1. Cross-Linker Selection

The first part of this study focused on the preliminary selection of two different cross-linkers: a trifunctional cross-linker, trimethylolpropane triacrylate (TRIM), and a bifunctional one, ethylene glycol dimethacrylate (EGDMA). These were evaluated by combining them with two commonly employed monomers, 2-vinylpyridine (2VP) and methacrylic acid (MAA), which are frequently reported in the literature [9,13,18,19,20].

**Figure 4:**
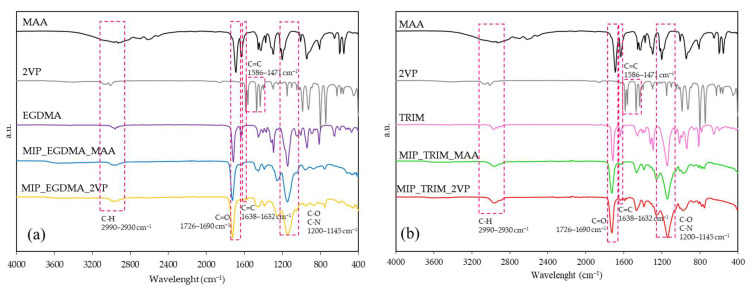
FT-IR spectra of reagents and corresponding MIPs: (**a**) MIPs prepared with EGDMA; (**b**) MIPs prepared with TRIM.

Figure 4 shows the FT-IR spectra of the synthesized MIPs together with the corresponding monomers and cross-linkers. The unsaturation of the double bonds (C=C), set at 1634–1638 cm^−1^, for MAA and the cross-linkers, respectively, is disappearing or significantly diminishing, indicating the reaction evolution [24,25,26,27]. In the case of 2VP, the vinylic group unsaturation corresponds to the peaks at 1586 cm^−1^ and 1471 cm^−1^, while other absorptions at 1563 and 1463 cm^−1^ are attributed to the aromatic pyridine ring [28]. In general, the spectra exhibit the presence of important bands set at 1200–1145 cm^−1^ (C-O and C-N stretch), 1728 cm^−1^ (C=O stretch), and 2990–2930 cm^−1^ (C-H stretch). Control NIPs were also synthesized, but no significant spectral differences were observed compared to the MIPs.

**Figure 5:**
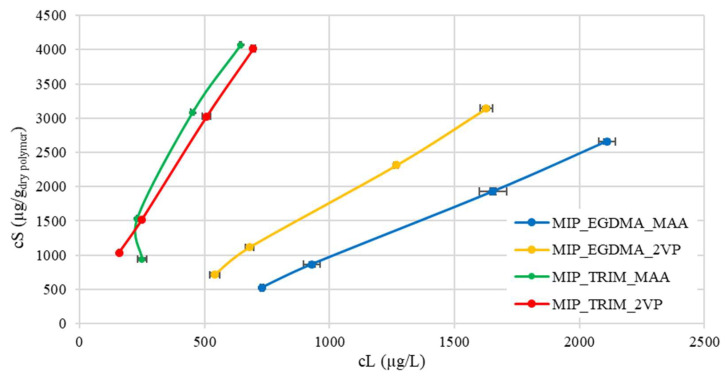
Isotherms of MIPs prepared with EGDMA and TRIM cross-linkers, conducted with deionized water.

The adsorption capacity of the materials was evaluated in synthetic solutions prepared with deionized water. The adsorption data are presented as isotherms, where the concentration of CBZ adsorbed by the material (cS) is plotted against the concentration remaining in the solution (cL) once equilibrium is reached (Figure 5). The CBZ adsorbed concentration was calculated according to Equation (1).

(1) $$cS=\frac{\left(cL0-cL\right)V}{m}$$

where cL0 is the initial concentration of the spiked solution (mg/L), V is the liquid volume (L), and m is the mass of the polymer (g).

The data obtained from the isotherms show that the trifunctional cross-linker offers the best results, with a higher ratio of adsorbed concentration to concentration remaining in solution. This trend can also be attributed to the greater surface area of these materials, as demonstrated by the BET analyses reported in Table 2. The increased available surface area, due to the trifunctional cross-linker, appears to favor adsorption. The surface areas of MIP_EGDMA_2VP (270 m^2^/g) and MIP_TRIM_MAA (440 m^2^/g) are consistent with values reported by other authors [16] but significantly higher than those reported by Dai et al. [9] and Esfandyari-Manesh et al. [29], equal to 136 m^2^/g and 242 m^2^/g, respectively. These values are also much higher than the surface area obtained using ESOA (epoxidized soybean oil acrylate) as the cross-linker, which was found to be close to 0 m^2^/g [16]. Notably, in the MIP reported by Dai et al. [9], the monomer-to-cross-linker ratio was 1:5.4, while in the one reported by Esfandyari-Manesh [29], it was 1:1. This suggests that the conditions employed in the synthesis enhance the porosity of the materials.

**Figure 6:**
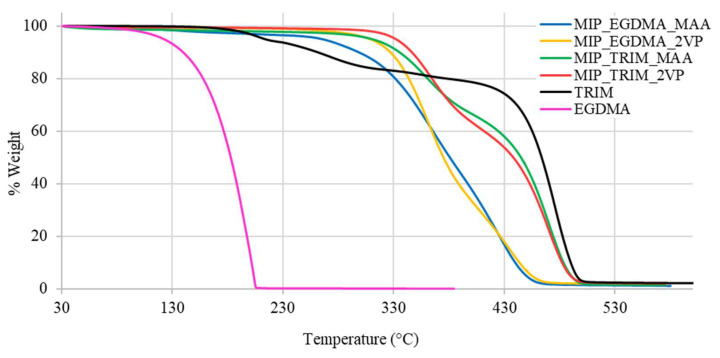
TGA thermograms of MIPs prepared with EGDMA and TRIM cross-linkers compared to those of pure cross-linkers.

The effect of the trifunctional cross-linker is also reflected in the thermal stability of the materials determined by thermogravimetric analysis (TGA), which is a technique that monitors the mass of a sample continuously as a function of temperature under controlled atmosphere. This method provides detailed quantitative information on thermal degradation behavior, including decomposition onset temperature (T_onset_) and the number and nature of distinct degradation steps, which are particularly significant in crosslinked polymer networks. As shown by the thermograms reported in Figure 6, the MIPs synthesized with TRIM start degrading at higher temperatures and show two distinct degradation steps, reflecting the thermal profile of the starting reactants.

The analysis of the MIP T_onset_ shows the following order of thermal stability: MIP_TRIM_2VP > MIP_TRIM_MAA ≈ MIP_EGDMA_2VP > MIP_EGDMA_MAA (Table 3). The presence of the trifunctional cross-linker promotes the formation of a more compact polymer network, resulting in higher thermal stability. In the case of the monomer, 2VP, possessing an aromatic ring, it starts degrading at higher temperatures with respect to MAA. Therefore, it is assumed that the presence of 2VP contributes to an even higher thermal stability of the corresponding MIP.

The variations in thermogram profiles further indicate that polymerization has taken place and that the choice of monomer affects the outcome.

### 2.2. Bio-Based Monomers Selection

The six selected monomers (2-hydroxyethyl methacrylate (HEMA), ferulic acid (FER), fumaric acid (FUM), eugenol (EUG), coumaric acid (COU), 3-(furyl)acrylic acid (FAA)) were used to produce MIPs, with the trifunctional cross-linker, which proved to be the best in the preliminary tests. All the results were also compared with MIP_TRIM_MAA previously prepared.

**Figure 7:**
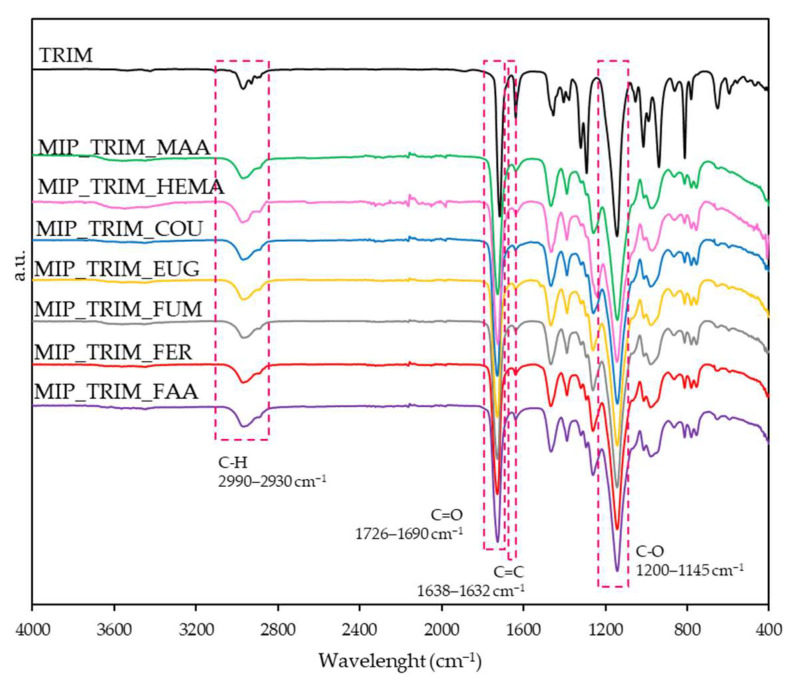
FT-IR spectra of MIPs with bio-based monomers and TRIM.

The FT-IR analyses, shown in Figure 7, indicate the reaction evolution by the significant lowering of the signal corresponding to the C=C double bonds at 1638 cm^−1^. Other significant transmittance bands, common to all the materials prepared, are the C=O double bond stretch at 1728 cm^−1^ and the C-H stretching at 2997 cm^−1^. The polymer’s carbonyl group is shifted to higher wavenumbers with respect to the carbonyl of the α,β-unsaturated ester present in TRIM (1716 cm^−1^). This shift suggests that the double bond of the cross-linkers reacted, producing an aliphatic ester.

A semi-quantitative elaboration was also conducted evaluating the ratio between the C=C and C=O height. As reported in Table 4, this ratio sensibly decreases with respect to the cross-linker.

**Figure 8:**
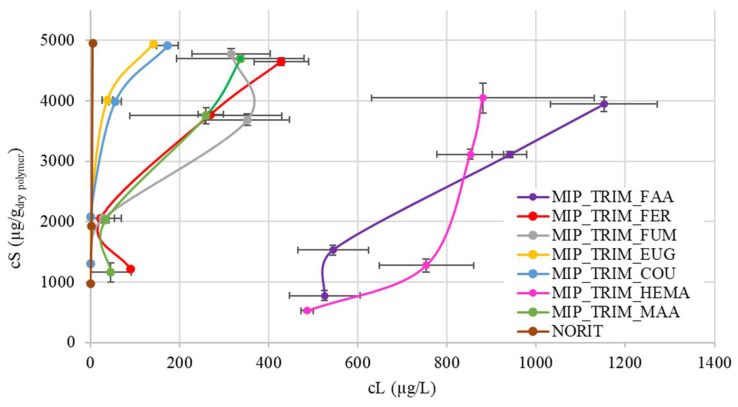
CBZ adsorption isotherms of MIPs with bio-based monomers, conducted with actual WWTP effluent spiked with CBZ.

The CBZ adsorption capacity of the materials was evaluated by spiking the wastewater treatment plant (WWTP) effluent (Table 5) with CBZ to consider the matrix effect. A commercial material, activated carbon Norit, is also reported as a reference. The adsorption data can be divided into three main groups (Figure 8). An enlargement of Figure 8 with detailed plots relative to the three groups of identified MIPs is reported in the Supporting Information (Figures S1–S3).

The first group (Figure S1) includes polymers with COU and EUG. Both materials provide the best results, likely due to the presence of the aromatic ring, which forms π-π stacking with CBZ. In the case of EUG, the aromatic ring has multiple substituents, and the unsaturation, which connects the monomer to the lattice, is one carbon away, allowing for greater movement of the ring. In the case of COU, the aromatic ring is sterically less hindered, with only one substituent, while the mobility of the chain connecting to the lattice is reduced since the double bond is one position away from the ring. Additionally, COU has an acidic group that may favor interaction with CBZ through hydrogen bonding. These two materials exhibit high adsorption capacity at low concentrations, comparable to activated carbon (Norit).

The second group (Figure S2) includes MAA, FUM, and FER. FUM exhibits lower adsorption due to the lack of an aromatic ring. In the case of FER, despite its structure being quite similar to EUG, it is hypothesized that the presence of the methoxy group and the limited mobility of the aromatic ring may lead to a more challenging arrangement of the molecule around CBZ during adsorption.

Finally, the third group (Figure S3) includes the MIPs synthesized with HEMA and FAA. HEMA shows the lowest adsorption, confirming that interaction with CBZ is due to acid group or π-π interaction. In the case of FAA, the low adsorption may be attributed to the poor wettability of the resulting material. Furthermore, FAA could inhibit radical polymerization or react with CBZ through a Diels–Alder reaction [30,31].

To further demonstrate the strong interaction between CBZ and EUG, a study on peak shifts due to their interaction was conducted using both FT-IR and UV analysis [32]. In the FT-IR test, CBZ was solubilized in EUG, while in the UV test, both molecules were dissolved in water to confirm that the interaction also occurred in the presence of water (Supporting Information, Text S1 and Figures S4–S7).

The thermal analysis confirms that the presence of bio-based monomers does not affect the materials’ stability, since they remain stable up to 300 °C, as shown in Table 6.

**Figure 9:**
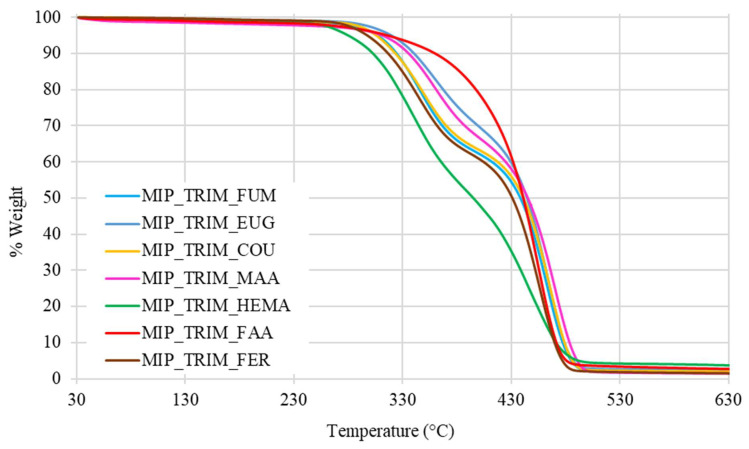
Thermograms of MIPs with different bio-based monomers.

The thermograms of MIPs with FUM, EUG, COU, MAA, and FER show a pattern like that of the MIPs previously synthesized with the same type of cross-linker, featuring a two-step degradation process. In the case of the MIP with FAA, the thermogram displays a single-step degradation pattern with a higher onset of degradation temperature due to interactions involving FAA during the polymerization process. Finally, for the MIP with HEMA, a degradation trend at lower temperatures is observed, probably ascribable to the absence of aromatic rings and acidic groups capable of forming hydrogen bonds and stabilizing the network (Figure 9).

### 2.3. Reaction Conditions Optimization

Considering the results obtained from the monomer selection, we decided to further investigate the EUG-based MIPs.

As reported by Pratama et al. [33], the ratio between the amount of monomer and cross-linker used in the synthesis of MIP is one of the factors influencing the material’s adsorptive capabilities. For this reason, the behavior of a MIP with the same reagents but a higher percentage of monomer was evaluated. Another factor examined was the reaction time, which was extended from 4 h to 24 h. For each material, a reference polymer (NIP, not imprinted polymer) synthesized without the addition of the template molecule (CBZ) was also prepared.

**Figure 10:**
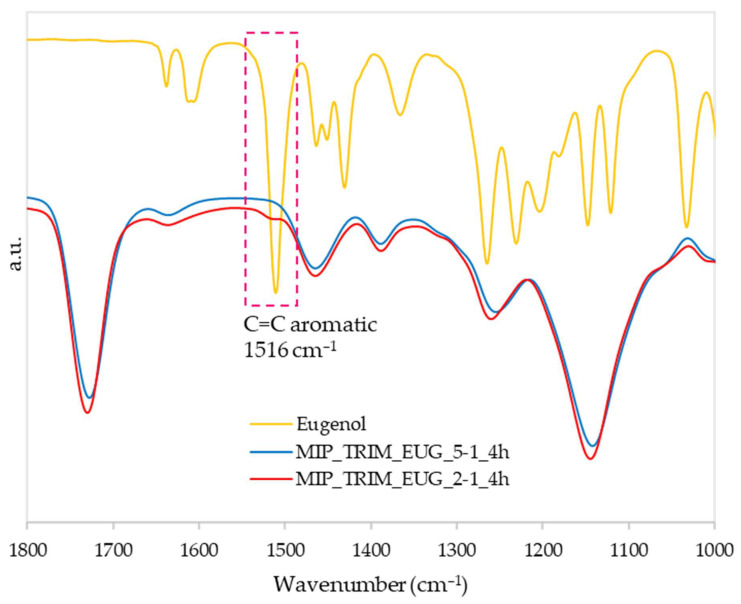
FT-IR spectra of MIP with cross-linker/monomer ratio 5/1 (MIP_TRIM_EUG_5-1_4h) and the one with cross-linker/monomer ratio 2/1 (MIP_TRIM_EUG_2-1_4h).

As in the previous test, FT-IR analyses confirmed the reaction evolution (spectra not reported), indicating no differences among the samples prepared with different reaction times. Conversely, in the case of the sample with a higher EUG concentration (MIP_TRIM_EUG_2-1_4h), a small but not negligible shoulder was detected at 1516 cm^−1^, attributed to the stretching of the aromatic ring; this peak confirms the presence of the monomer (Figure 10) [34].

Further adsorption isotherms were conducted using the WWTP effluent enriched not only with CBZ but also with diclofenac (DCF) and ibuprofen (IBU) to evaluate both the CBZ adsorption capacity and the selectivity towards CBZ. CBZ, DCF, and IBU have similar structures with different steric hindrances. Higher initial pharmaceutical concentrations were tested (25, 50, 100 mg/L) in comparison to the previous tests, to verify more accurately MIP selectivity towards CBZ.

**Figure 11:**
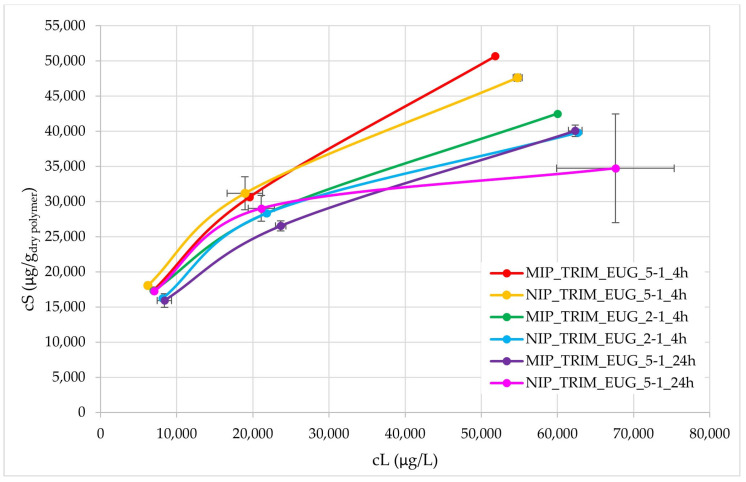
Adsorption isotherms of MIPs and NIPs synthesized with EUG in different reaction conditions, conducted with actual WWTP effluent spiked with CBZ, DCF, and IBU.

The resulting isotherms are shown in Figure 11. In the initial part, up to adsorbed concentrations (*cS*) of 3 mg/g, the isotherms are generally overlapping, except for MIP_TRIM_EUG_5-1_24h, which deviates and exhibits lower adsorption. At higher concentrations, the best performance is observed for MIP_TRIM_EUG_5-1_4h and NIP_TRIM_EUG_5-1_4h, whereas the material with the worst adsorption performance is NIP_TRIM_EUG_5-1_24h. In all cases, at high concentrations, a trend is observed that confirms the superior adsorption performance of the MIP compared to the corresponding NIP. It is also evident that the adsorption isotherm of MIP_TRIM_EUG_5-1_4h is higher than that of MIP_TRIM_EUG_2-1_4h. This result confirms that the molar ratio composition influences the performance of the final materials. Increasing the amount of monomer affects the adsorption capacity. A possible explanation could be the ambivalent effect of EUG, which shows an interaction with CBZ, thus favoring complex formation, but it may also slow down the polymerization process.

The difference between MIP and NIP was also assessed through the ratio of adsorption capacities, and no significant differences were observed at the two lowest concentrations.

This result can be attributed to the fact that the adsorption of CBZ in the NIP is primarily driven by a surface adsorption process, characterized by a strong interaction between the aromatic ring of eugenol and CBZ. Since no CBZ molecules were introduced during the synthesis of NIP to act as templates for the formation of selective cavities, the polymer lacks specific recognition sites. However, the presence of a monomer with a high affinity for CBZ still facilitates a generalized adsorption of the compound. In order to better investigate the differences between MIP and NIP, a kinetic test of CBZ adsorption on MIP_TRIM_EUG_5-1_4h and NIP_TRIM_EUG_5-1_4h was conducted, monitoring the CBZ concentration every minute for the first 10 min and then every 10 min, and the calculated solid-phase concentrations were interpolated with both a first-order and second-order kinetic model. As shown in Figure S8 in the Supplementary Information, during the initial 60 min, CBZ adsorption on MIP_TRIM_EUG_5-1_4h was significantly faster than on NIP_TRIM_EUG_5-1_4h. This finding indicates that in the case of a scale-up process in continuous flow conditions, MIP_TRIM_EUG_5-1_4h would require a significantly shorter contact time than NIP_TRIM_EUG_5-1_4h, which implies a reduction both in the column investment cost and in the operational cost for the periodic sorbent replacement.

**Figure 12:**
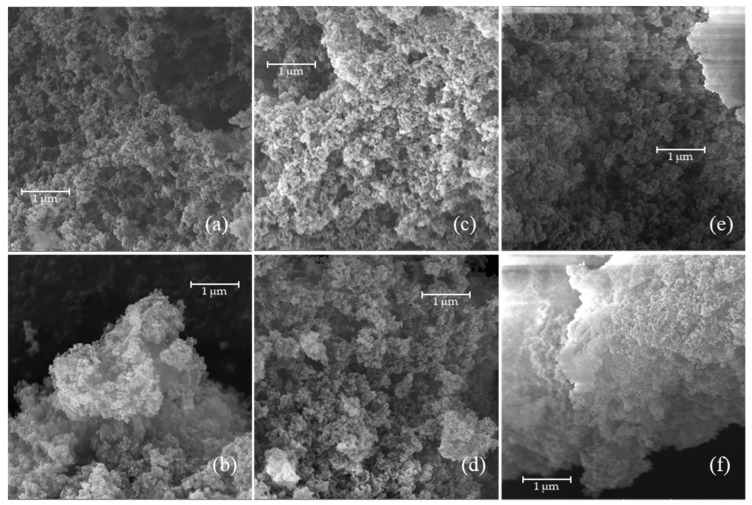
SEM pictures of MIPs at a resolution of 40kx: (**a**) MIP_TRIM_EUG_5-1_4h; (**b**) NIP_TRIM_EUG_5-1_4h; (**c**) MIP_TRIM_EUG_2-1_4h; (**d**) NIP_TRIM_EUG_2-1_4h; (**e**) MIP_TRIM_EUG_5-1_24h; and (**f**) NIP_TRIM_EUG_5-1_24h.

The MIPs prepared were also characterized in terms of morphology by scanning electron microscopy (Figure 12), and the surface area was evaluated using a BET.

The morphology of MIPs primarily depends on the synthesis method used, followed by the choice of porogen, and finally the cross-linker [16,35]. Typically, polymers synthesized via precipitation polymerization exhibit homogeneous, spherical particles, while those prepared by bulk polymerization display more heterogeneous structures [36,37]. SEM images of MIPs prepared by bulk polymerization reveal typical morphologies characterized by agglomerates of irregularly shaped particles of varying sizes, often described as having a cauliflower-like appearance [37].

The SEM analysis was conducted to assess whether increasing the monomer concentration and extending the reaction time would affect the polymer structure formation. The results show that the morphologies obtained are extremely porous and comparable, indicating that neither the monomer-to-cross-linker ratio nor the reaction duration significantly influence the MIP morphology. This conclusion is further supported by the similarities in surface area values reported in Table 7, indicating that the investigated reaction conditions do not have a high impact on the sample morphology.

The MIP surface areas were slightly lower than those of NIPs, suggesting the possible presence of a residual template within the MIPs. Furthermore, the highest surface area was exhibited by the MIP synthesized with a lower EUG concentration and a longer reaction time. However, this sample did not demonstrate the best adsorption capacity; therefore, it can be concluded that the differences in adsorption capacity cannot be attributed to morphological variations. The MIP and NIP surface areas were equal to about 50% of the area of a typical activated carbon (Norit GAC 1240W).

**Figure 13:**
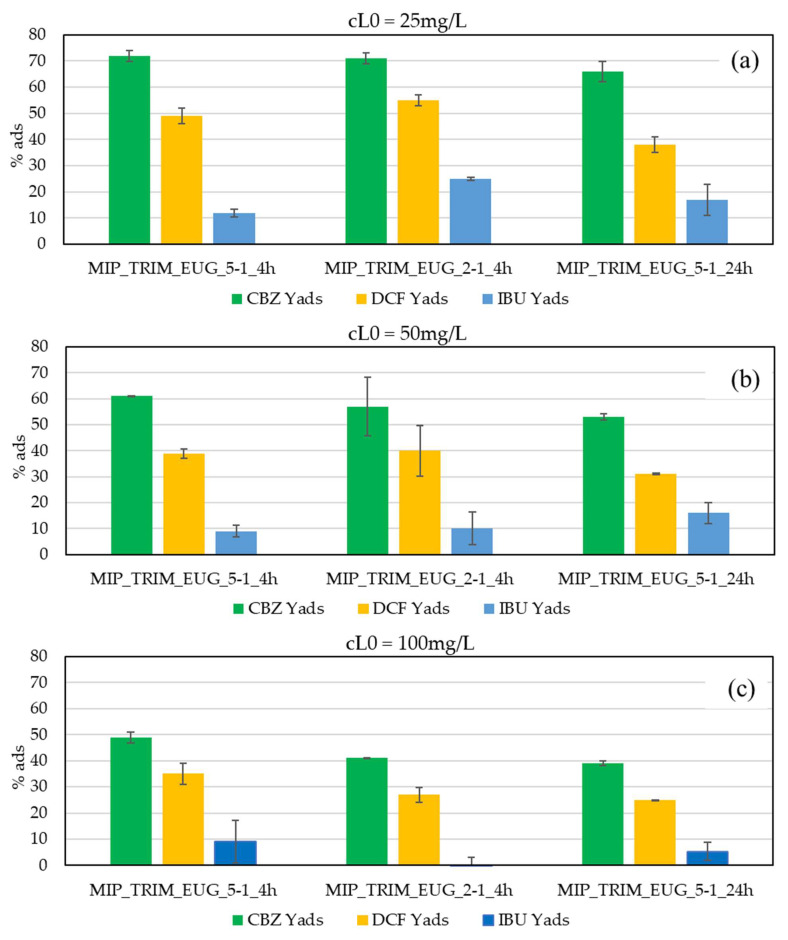
Analysis of the selectivity of three types of MIPs with EUG for CBZ, DCF, and IBU. Tests were conducted with actual WWTP effluent.

The results of the test of MIP_TRIM_EUG selectivity for CBZ compared to other pharmaceuticals are presented in Figure 13. The percentage of CBZ adsorbed by MIPs decreases with the rise in cL0, with adsorption values of 70%, 57%, and 43%, respectively, for initial concentrations of 25 mg/L, 50 mg/L, and 100 mg/L. In the literature, the adsorption capacity of MIPs for CBZ solutions in distilled water without interfering substances exceeds 90% [13,20]. However, in the case of solutions in distilled water containing multiple drugs, the adsorption capacity drops to values as low as 40% [3]. These data suggest that multiple pollutants’ presence in the considered solution and the type of water used significantly affect the overall adsorption capacity of the materials.

In terms of adsorption yield (Y_ads_) relative to CBZ, DCF and IBU were obtained at different initial concentrations in the 25–100 mg/L range. The Y_ads,CBZ_/Y_ads,DCF_ ratio was equal to 1.5 ± 0.1, whereas the Y_ads,CBZ_/Y_ads,IBU_ ratio was equal to 6 ± 3, indicating a high selectivity of the tested TRIM_EUG MIPs towards CBZ. The selectivity did not vary significantly among the three MIPs based on TRIM and EUG.

Considering the results, increasing the reaction time did not prove to be an effective choice. Since similar adsorption capacities and comparable morphological profiles were obtained, it is preferable to maintain a reaction time of 4 h with a view to future scale-up. This would also help to reduce energy consumption and production costs.

TGA revealed a comparable degradative behavior between the MIPs and NIPs prepared under identical conditions. This result indicates that the presence of the template during the synthesis process does not affect the thermal stability of the polymer network.

**Figure 14:**
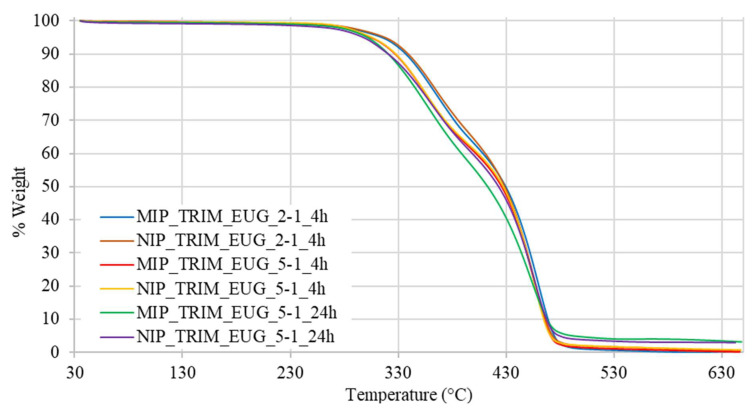
TGA thermograms of MIPs with different reaction conditions.

As illustrated in Figure 14 and Table 8, the materials synthesized with an increased monomer-to-cross-linker ratio exhibit a T_onset_ approximately 10 °C higher than that prepared with a lower one. This suggests that a higher monomer content enhances the material’s stability, likely due to the aromatic group present in EUG.

### 2.4. MIP Recyclability and Performances

**Figure 15:**
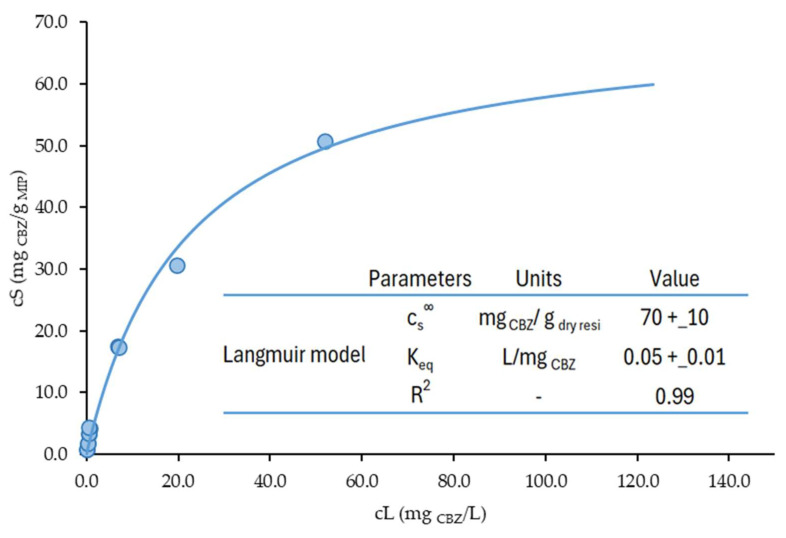
Complete CBZ adsorption isotherm of MIP_TRIM_EUG_5-1_4h, best-fitting Langmuir simulation, and corresponding best estimates of the model parameters, with 95% confidence intervals.

For the material that demonstrated the best performance, MIP_TRIM_EUG_5-1_4h, a more comprehensive study was carried out to evaluate its behavior at low concentrations of CBZ and to determine the saturation limit. The resulting isotherm is presented in Figure 15, and the data were subsequently fitted using the Langmuir model, as expressed in Equation (2):

(2) $$c_{s}=\frac{c_{s}^{\infty }c_{L}}{\frac{1}{K_{eq}}+c_{L}}$$

where $c_{s}^{\infty }$ (mg_CBZ_/g_dry resin_) is the maximum amount sorbed per unit mass of adsorbent, corresponding to a complete monolayer on the adsorbent surface, and K_eq_ (L/mg_CBZ_) is the constant related to the affinity between the binding sites and CBZ. The best-fit parameters obtained are reported in the box in Figure 15 with their 95% confidence interval. The quality of the fitting was evaluated by calculating the coefficient of determination R^2^, that resulted in being equal to 0.99.

**Figure 16:**
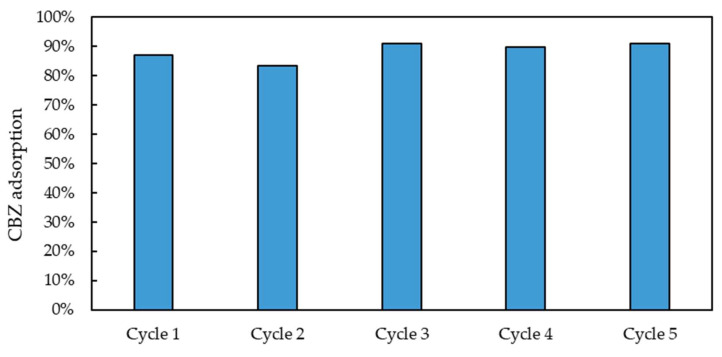
Adsorption of CBZ on MIP_TRIM_EUG_5-1_4h during 5 consecutive adsorption–desorption cycles.

As shown in Figure 15, eight additional isotherm points were tested in the 70–740 μg/L liquid-phase concentration range at equilibrium to investigate the CBZ adsorption performances of MIP_TRIM_EUG_5-1_4h under conditions closer to those of real applications. The sorption performance of MIP_TRIM_EUG_5-1_4h at very low CBZ levels typical of WWTP (0.1–38 μg/L, average value 4.8 μg/L [38]) can be safely estimated by extrapolating the strongly linear trend presented by these eight additional points tested in the low concentration range (R^2^ = 0.963). An additional study was carried out to assess the recyclability of the material. Five adsorption–desorption cycles were carried out using a WWTP effluent spiked with CBZ at 4 mg/L. For the desorption step, a methanol/acetic acid solution (9:1), also used for template removal post-polymerization, was employed. The set-up and procedure of the repeated adsorption–desorption tests are reported in Section 3.3. As illustrated in Figure 16, the material maintains a high adsorption capacity (about 90%, in line with MIPs synthesized for other pharmaceuticals [39]) during the repeated cycles. In particular, no significant differences were observed in the adsorption capacities obtained during the five repeated cycles (*t* test, *p* = 0.05). Furthermore, the amount of desorption solution needed to completely regenerate the sorbent was very low (5–10 bed volumes), as reported in the Supporting Information, Figure S9. The stability of the sorbent performances over multiple cycles and the low amount of regenerant required allowed for the achievement of a relevant reduction in the operating cost associated with the periodic regeneration and replacement of the sorbent.

The synthesized material demonstrates excellent reusability, showing a performance comparable to that of other CBZ-targeting MIPs described in the literature [9,19,32].

Furthermore, the study of MIP_TRIM_EUG_5-1_4h over a broader concentration range allowed for a comparison with other materials reported in the literature, such as the resveratrol-selective MIP synthesized using a bio-based cross-linker [16]. Notably, at the same liquid-phase equilibrium concentration (C_e_ = 28 mg/L), the MIP developed in this study is capable of adsorbing a significantly higher amount of analyte (35 mg/g of CBZ versus 3 mg/g of resveratrol).

Additionally, the slope of the initial portion of the CBZ adsorption isotherm obtained with MIP_TRIM_EUG_5-1_4h (0.007 L/mg) can be compared with that of other MIPs designed for CBZ, such as the materials synthesized via precipitation polymerization and deposited onto magnetic carriers (Fe_3_O_4_@SiO_2_) [32]. In that case, the slope varies depending on the monomer used, ranging from 0.08 to 0.002 L/mg. It should be noted, however, that the synthesis conditions differ between systems.

At the same equilibrium concentration (50 mg/L), the CBZ-selective MIP developed in this study adsorbs 50 mg/g, while MIPs prepared for IBU, under comparable conditions, reach only 7.2 mg/g [36]. Although differences in target molecule properties may influence the adsorption behavior, these results clearly highlight the superior performance of the CBZ-specific MIP developed in this study in terms of adsorption capacity [36].

## 3. Materials and Methods

### 3.1. Materials

Pharmaceuticals: Diclofenac sodium salt (DCF, >98.0%), ibuprofen (IBU, >98.0%), and carbamazepine (CBZ, >97.0%) were all purchased from Tokyo Chemical Industry Co., Ltd. (Tokyo, Japan).

3-(2-furyl) acrylic acid (FAA, 99%) was purchased from Sigma-Aldrich (now Merk, St. Louis, MO, USA). Methacrylic acid (MAA, >99%), 2-vinylpyridine (2VP, 97%), 2-hydroxyethymethacrylate (HEMA, 97%), ferulic acid (FER, ≥99%), eugenol (EUG, 99%), ethylene glycol dimethacrylate (EGDMA, 98%), trimethylolpropane triacrylate (TRIM), methanol (MeOH, ≥99.8%), acetic acid (AA, ≥99.8%), and acetonitrile (ACN, 98%) were all purchased from Sigma Aldrich. Azobisisobutyronitrile (AIBN, ≥98%), fumaric acid (FUM, ≥99%), and coumaric acid (COU, ≥98%) were purchased from Fluka Chemie (Buchs, Switzerland). All reagents were used as received without any further purification. Norit GAC 1240 W was purchased from Norit Italia Spa (Ravenna, Italy). The wastewater treatment plant (WWTP) effluent samples were provided from a WWTP located in Northern Italy. The plant’s treatment train includes a typical activated sludge section with pre-denitrification, secondary settling, and chemical disinfection. The average effluent composition is reported in Table 5.

### 3.2. Preparation of MIPs

Materials were prepared by bulk polymerization, adapting the procedure reported by Cantarella et al. [23]. A typical reaction protocol involves the mixture of 2.0 (or 4.0) mmol of monomer (MAA, 2VP, HEMA, FER, FUM, COU, or EUG), 0.2 mmol of template (CBZ), and 8 mL of porogen (ACN) in a three-necked flask for 20 min. This step allows for a better interaction between the monomer and the template, allowing for complex formation. Then, 10 (or 8) mmol of cross-linker (EGDMA or TRIM) and 5.1 mmol of initiator (AIBN) are added. The reaction mixture is degassed with a flow of N_2_ for 5 min, sealed under N_2_, and placed in a silicon oil bath at 70 °C for 4 h (or 24 h) to carry out the polymerization process. The resulting bulk polymer is crushed in a mortar. Finally, the template and the non-polymerized compounds are extracted by washing the polymer powders through the following steps: three subsequent incubations of 30 min in 50 mL of a MeOH/AA solution (9:1, *v*/*v*), and one incubation with just MeOH to remove acetic acid. The complete removal of CBZ was confirmed by analyzing the washing effluent with HPLC-DaD (see Supporting Information, Figure S9). The polymer is then dried overnight at 65 °C with vacuum. Reference materials, called NIP (not imprinted polymer), were produced without the addition of the template.

### 3.3. Characterization of MIPs

Thermogravimetric analysis (TGA) was performed with a PerkinElmer Thermogravimetric Analyzer TGA4000 (PerkinElmer, Inc., Hopkinton, MA, USA) using a ceramic pan. Tests were conducted in a nitrogen atmosphere with a flow rate of 20 mL/min; the heating rate was 10 °C/min from 35 °C to 650 °C. The samples weighed around 10 mg.

FT-IR analysis was conducted with a PerkinElmer FT-IR Spectrum 3 spectrometer (PerkinElmer, Inc., Hopkinton, MA, USA). All spectra were recorded after 16 scans over the wavenumber range of 400–400 cm^−1^.

Preliminary batch isotherm tests to select the cross-linker were performed in synthetic solutions prepared with deionized water. Initial concentrations of CBZ ranged from 1 to 5 mg/L, with an adsorbent material concentration of 1 g/L. The rotary shaker was set to a temperature of 22 °C and a rotational speed of 160 rpm for 24 h. Batch isotherm tests to select the monomer were performed in real WWTP effluent under the same conditions as described above. For the best monomer, isotherm tests to analyze the best reaction conditions were performed in real WWTP effluent spiked with CBZ, IBU, and DCF with initial concentration ranging from 25 to 100 mg/L. Experimental details on the isotherm tests are described by Pinelli et al. [40]. The adsorption–desorption repeated tests on the best selected sorbent, MIP_TRIM_EUG_5-1_4h, were conducted in a small column that was packed following the Rohm and Haas procedure, reaching 9 cm of bed height [41]. The adsorption was conducted in recycle mode, starting from a solution containing 4 mg/L of CBZ that was fed to the column and then recirculated to the inlet becher. After 24 h, the liquid phase was in equilibrium with the sorbent, and by measuring the equilibrium concentration in the liquid, the adsorbed mass was calculated. Desorption was conducted in continuous mode with MeOH acidified with 10% AA at an empty bed contact time (EBCT) of about 15 min. After each adsorption or desorption step, a rinsing step was performed. The CBZ, DCF, and IBU content was determined using HPLC-DaD Agilent Infinity 1260. The method used for pharmaceutical quantification employs a 1 mL/min flux of the mobile phase composed of MeOH:H_2_O 70:30 *v*/*v* with 0.1% AA, an injection volume of 20 µL, and an Agilent Zorbax C18 Eclipse Plus column. The materials’ surface area was determined by Brunauer–Emmett–Teller analysis using a Quantachrome Nova 2200e instrument (Anton Paar Quanta, Graz, Austria), with N_2_ at 77 K used as the gas.

SEM analyses were performed using a Tescan Mira 3 field-emission scanning electron microscope (FESEM) (TESCAN Group a.s., Kohoutovice, Czech Republic). Samples were sputter-coated with a thin layer of gold prior to analysis.

## 4. Conclusions

This study shows that molecular imprinted polymers (MIPs) represent a promising class of materials for the selective adsorption of carbamazepine (CBZ) from real wastewater treatment plant (WWTP) effluents. The results highlight the importance of cross-linker and monomer selection in developing MIPs for the selective removal of CBZ from aqueous solutions. The trifunctional cross-linker (TRIM) proved to be the most effective, yielding materials with enhanced adsorption capacity, thermal stability, and a surface area twice as high as those obtained with the bifunctional cross-linker. Among the bio-based monomers tested, eugenol (EUG) and coumaric acid (COU) exhibited the highest affinity for CBZ due to their aromatic structures, which facilitate π-π interactions.

Further optimization of the reaction conditions demonstrated that increasing the monomer-to-cross-linker ratio (MIP_TRIM_EUG_2-1_4h) improves the imprinting capacity of the material. However, the highest CBZ adsorption was observed for MIP_TRIM_EUG_5-1_4h, suggesting that a higher monomer concentration creates more specific active sites for CBZ but also hinders its release. For MIP_TRIM_EUG_5-1_4h, the adsorption capacity was further evaluated by fitting the isotherm data to the Langmuir model, confirming monolayer adsorption behavior. Additionally, the reusability of the material was tested, showing that it could undergo up to five adsorption–desorption cycles without any significant loss in performance. Additionally, thermal analysis confirmed that the synthesized materials remain stable up to 300 °C.

These findings contribute to the advancement of sustainable MIP-based materials for wastewater treatment, offering an environmentally friendly approach to removing pharmaceutical contaminants. Future studies should investigate the scalability of these materials to further validate their practical applicability.

## Figures and Tables

**Table 1:** Composition, reaction conditions, and codes of the materials prepared.

Template	Cross-Linker	Monomer	Cross-Linker /Monomer Molar Ratio	Reaction Time (h)	Sample Code
CBZ	EGDMA	MAA	5/1	4	MIP_EGDMA_MAA
		2VP			MIP_EGDMA_2VP
	TRIM	MAA			MIP_TRIM_MAA
		2VP			MIP_TRIM_2VP
CBZ	TRIM	EUG	5/1	4	MIP_TRIM_EUG
		MAA			MIP_TRIM_MAA
		COU			MIP_TRIM_COU
		FUM			MIP_TRIM_FUM
		FER			MIP_TRIM_FER
		FAA			MIP_TRIM_FAA
		HEMA			MIP_TRIM_HEMA
/	TRIM	EUG	5/1	4	NIP_TRIM_EUG_5-1_4h
CBZ			2/1	4	MIP_TRIM_EUG_2-1_4h
/			2/1	4	NIP_TRIM_EUG_2-1_4h
CBZ			5/1	24	MIP_TRIM_EUG_5-1_24h
/			5/1	24	NIP_TRIM_EUG_5-1_24h

**Table 2:** Specific surface area of MIPs prepared with EGDMA, and TRIM cross-linkers, determined by BET analysis.

Sample	Specific Surface Area (m^2^/g)
MIP_EGDMA_MAA	210
MIP_EGDMA_2VP	270
MIP_TRIM_MAA	440
MIP_TRIM_2VP	530

**Table 3:** TGA data of onset temperatures and 5% weight loss temperatures of the tested MIPs.

	T_onset_ (°C)	T_5%_ (°C)
MIP_EGDMA_MAA	308	266
MIP_EGDMA_2VP	327	309
MIP_TRIM_MAA	322	311
MIP_TRIM_2VP	333	332

**Table 4:** Ratio between double bond and carbonyl signals in FT-IR spectra.

Sample	h_C=C_^a^	h_C=O_^b^	h_C=C_/h_C=O_
MIP_TRIM_COU	2.11	31.43	0.07
MIP_TRIM_EUG	1.95	27.64	0.07
MIP_TRIM_FER	2.03	25.45	0.08
MIP_TRIM_FUM	1.93	25.04	0.08
MIP_TRIM_MAA	3.22	33.18	0.10
MIP_TRIM_HEM	1.22	13.41	0.09
MIP_TRIM_FAA	2.44	26.56	0.09
TRIM	24.28	75.38	0.32

^a^ calculated at 1638 cm^−1^; ^b^ calculated at 1728 cm^−1^.

**Table 5:** Compositions of the WWTP effluent used for the isotherm tests (average values ± 95% confidence intervals).

Compound	Symbol	Unit	Bologna TMWW
Biological oxygen demand	BOD_5_	mg_O2_ L^−1^	15 ± 2
Chemical oxygen demand	COD	mg_O2_ L^−1^	29 ± 2
Total suspended solids	TSS	mg L-1	5.3 ± 0.6
Ammonium nitrogen	NH_4_-N	mg_N_ L^−1^	3.9 ± 0.3 a
Potassium	K^+^	mg L^−1^	13 ± 2
Magnesium	Mg^2+^	mg L^−1^	14 ± 1
Sodium	Na^+^	mg L^−1^	101 ± 8
Calcium	Ca^2+^	mg L^−1^	83 ± 6
Chloride	Cl^−^	mg L^−1^	150 ± 12
Nitrate	NO_3_^−^	mg_N_ L^−1^	6.7 ± 0.5
Phosphate	PO_4_-P	mg_P_ L^−1^	1.0 ± 0.2
Sulfate	SO_4_^2–^	mg L^−1^	104 ± 9
pH	pH	-	7.9 ± 0.2

**Table 6:** TGA data of onset temperatures and 5% weight loss temperatures of MIPs with bio-based monomers.

Sample	T_onset_ (°C)	T_5%_ (°C)
MIP_TRIM_EUG	319	320
MIP_TRIM_MAA	322	311
MIP_TRIM_COU	307	306
MIP_TRIM_FUM	310	304
MIP_TRIM_FER	302	298
MIP_TRIM_FAA	409	316
MIP_TRIM_HEMA	294	280

**Table 7:** Specific surface area determined by BET analysis.

Materials	Specific Surface Area (m^2^/g)
MIP_TRIM_EUG_5-1_4h	437
NIP_TRIM_EUG_5-1_4h	465
MIP_TRIM_EUG_2-1_4h	489
NIP_TRIM_EUG_2-1_4h	494
MIP_TRIM_EUG_5-1_24h	560
NIP_TRIM_EUG_5-1_24h	460
NORIT GAC 1240W	1027

**Table 8:** TGA data of onset temperatures and 5% weight loss temperatures of MIPs with EUG.

Material	T_onset_ (°C)	T_5%_ (°C)
MIP_TRIM_EUG_5-1_4h	308	304
NIP_TRIM_EUG_5-1_4h	310	305
MIP_TRIM_EUG_2-1_4h	321	314
NIP_TRIM_EUG_2-1_4h	323	317
MIP_TRIM_EUG_5-1_24h	313	301
NIP_TRIM_EUG_5-1_24h	313	297

## Data Availability

Research data underlying this manuscript have been published in the Zenodo Research Repository (https://doi.org/10.5281/zenodo.15088793).

## Supplementary Materials

The following supporting information can be downloaded at: https://www.mdpi.com/article/10.3390/molecules30122533/s1, Figure S1: Adsorption isotherms of the first group of MIPs; Figure S2: Adsorption isotherms of the second group of MIPs; Figure S3: Adsorption isotherms of the third group of MIPs; Figure S4: FT-IR spectra of CBZ and EUG mixture and pure molecules; Figure S5: FT-IR spectra of CBZ and EUG mixture and pure molecules; Figure S6: FT-IR spectra of CBZ and EUG mixture and pure molecules; Figure S7: UV spectra of CBZ and EUG mixture and pure molecules; Figure S8: Kinetic adsorption study between the best performing MIP (MIP_TRIM_EUG_5-1_4h; Figure S9: Desorption curves obtained in 4 repeated adsorption/desorption tests on MIP_TRIM_EUG_%_1_4h; Figure S10: HPLC analysis of the washing solution for CBZ removal after polymerization; Table S1: Kinetic parameters for the best performing MIP (MIP_TRIM_EUG_5-1_4h. Text S1: Study of the interaction between CBZ and EUG. Text S2: Study on kinetic adsorption. Text S3: Study on MIP reusability. Text S4: Study on template removal.

## Abbreviations

The following abbreviations are used in this manuscript:

MIP	Molecularly imprinted polymer
NIP	Not imprinted polymer
NSAIDs	Non-steroidal anti-inflammatory drugs
CBZ	Carbamazepine
IBU	Ibuprofen
DCF	Diclofenac
EGDMA	Ethylene glycol dimethacrylate
TRIM	Trimethylolpropane triacrylate
MAA	Methacrylic acid
2VP	2-vinylpyridine
EUG	Eugenol
COU	Coumaric acid
FER	Ferulic acid
FUM	Fumaric acid
HEMA	2-hydroxyethyl methacrylate
FAA	3-(2-furyl) acrylic acid
TGA	Thermogravimetric analysis
WWTP	Wastewater treatment plant
EBCT	Empty bed contact time
MeOH	Methanol
AA	Acetic acid

## References

[1] Parlapiano M., Akyol C., Foglia A., Pisani M., Astolfi P., Eusebi A.L., Fatone F. (2021). Selective Removal of Contaminants of Emerging Concern (CECs) from Urban Water Cycle via Molecularly Imprinted Polymers (MIPs): Potential of Upscaling and Enabling Reclaimed Water Reuse. J. Environ. Chem. Eng..

[2] Pereira L.C., de Souza A.O., Bernardes M.F.F., Pazin M., Tasso M.J., Pereira P.H., Dorta D.J. (2015). A Perspective on the Potential Risks of Emerging Contaminants to Human and Environmental Health. Environ. Sci. Pollut. Res..

[3] Khulu S., Ncube S., Kgame T., Mavhunga E., Chimuka L. (2022). Synthesis, Characterization and Application of a Molecularly Imprinted Polymer as an Adsorbent for Solid-Phase Extraction of Selected Pharmaceuticals from Water Samples. Polym. Bull..

[4] Khan S., Naushad M., Govarthanan M., Iqbal J., Alfadul S.M. (2022). Emerging Contaminants of High Concern for the Environment: Current Trends and Future Research. Environ. Res..

[5] Wilkinson J.L., Boxall A.B.A., Kolpin D.W., Leung K.M.Y., Lai R.W.S., Galban-Malag C., Adell A.D., Mondon J., Metian M., Marchant R.A. (2022). Pharmaceutical Pollution of the World’s Rivers. Proc. Natl. Acad. Sci. USA.

[6] Adeyanju C.A., Ogunniyi S., Selvasembian R., Oniye M.M., Ajala O.J., Adeniyi A.G., Igwegbe C.A., Ighalo J.O. (2022). Recent Advances on the Aqueous Phase Adsorption of Carbamazepine. ChemBioEng Rev..

[7] European Parliament and Council (2024). Directive (EU) 2024/3019 of the European Parliament and of the Council OF 27 November 2024 Concerning Urban Wastewater Treatment (Recast) (Text with EEA Relevance).

[8] Rizzo L., Malato S., Antakyali D., Beretsou V.G., Đolić M.B., Gernjak W., Heath E., Ivancev-Tumbas I., Karaolia P., Lado Ribeiro A.R. (2019). Consolidated vs New Advanced Treatment Methods for the Removal of Contaminants of Emerging Concern from Urban Wastewater. Sci. Total Environ..

[9] Dai C.M., Zhang J., Zhang Y.L., Zhou X.F., Duan Y.P., Liu S.G. (2013). Removal of Carbamazepine and Clofibric Acid from Water Using Double Templates-Molecularly Imprinted Polymers. Environ. Sci. Pollut. Res..

[10] Mabrouk M., Hammad S.F., Abdella A.A., Mansour F.R. (2023). Tips and Tricks for Successful Preparation of Molecularly Imprinted Polymers for Analytical Applications: A Critical Review. Microchem. J..

[11] Sarpong K.A., Xu W., Huang W., Yang W. (2019). The Development of Molecularly Imprinted Polymers in the Clean-Up of Water Pollutants: A Review. Am. J. Analyt. Chem..

[12] Azizi A., Bottaro C.S. (2020). A Critical Review of Molecularly Imprinted Polymers for the Analysis of Organic Pollutants in Environmental Water Samples. J. Chromatogr. A.

[13] Kadhirvel P., Combès A., Bordron L., Pichon V. (2019). Development and Application of Water-Compatible Molecularly Imprinted Polymers for the Selective Extraction of Carbamazepine from Environmental Waters. Anal. Bioanal. Chem..

[14] Murdaya N., Triadenda A.L., Rahayu D., Hasanah A.N. (2022). A Review: Using Multiple Templates for Molecular Imprinted Polymer: Is It Good?. Polymers.

[15] Del Sole R., Mele G., Bloise E., Mergola L. (2021). Green Aspects in Molecularly Imprinted Polymers by Biomass Waste Utilization. Polymers.

[16] Le Goff N., Fomba I., Prost E., Merlier F., Haupt K., Duma L., Fayeulle A., Falcimaigne-Cordin A. (2020). Renewable Plant Oil-Based Molecularly Imprinted Polymers as Biopesticide Delivery Systems. ACS Sustain. Chem. Eng..

[17] Viveiros R., Rebocho S., Casimiro T. (2018). Green Strategies for Molecularly Imprinted Polymer Development. Polymers.

[18] Schweiger B., Bahnweg L., Palm B., Steinfeld U. (2009). Development of Molecular Imprinted Polymers (MIPs) for the Selective Removal of Carbamazepine from Aqueous Solution. World Acad. Sci. Eng. Technol..

[19] Dai C.M., Geissen S.U., Zhang Y.L., Zhang Y.J., Zhou X.F. (2010). Performance Evaluation and Application of Molecularly Imprinted Polymer for Separation of Carbamazepine in Aqueous Solution. J. Hazard. Mater..

[20] Mohiuddin I., Malik A.K., Aulakh J.S. (2020). Efficient Recognition and Determination of Carbamazepine and Oxcarbazepine in Aqueous and Biological Samples by Molecularly Imprinted Polymers. J. Anal. Chem..

[21] Sobiech M. (2024). Computer-Assisted Strategies as a Tool for Designing Green Monomer-Based Molecularly Imprinted Materials. Int. J. Mol. Sci..

[22] Gupta Y., Beckett L.E., Sadula S., Vargheese V., Korley L.S.T.J., Vlachos D.G. (2023). Bio-Based Molecular Imprinted Polymers for Separation and Purification of Chlorogenic Acid Extracted from Food Waste. Sep. Purif. Technol..

[23] Cantarella M., Carroccio S.C., Dattilo S., Avolio R., Castaldo R., Puglisi C., Privitera V. (2019). Molecularly Imprinted Polymer for Selective Adsorption of Diclofenac from Contaminated Water. Chem. Eng. J..

[24] Smith B.C. (2023). Infrared Spectroscopy of Polymers X: Polyacrylates. Spectroscopy.

[25] Lin-Gibson S., Bencherif S., Cooper J.A., Wetzel S.J., Antonucci J.M., Vogel B.M., Horkay F., Washburn N.R. (2004). Synthesis and Characterization of PEG Dimethacrylates and Their Hydrogels. Biomacromolecules.

[26] Lai W., Li X., Liu H., Han L., Zhao Y., Li X. (2014). Interfacial Polycondensation Synthesis of Optically Sensitive Polyurea Microcapsule. J. Chem..

[27] Zhang Y.Q., Yu P.L., Sun W.F., Wang X. (2021). Ameliorated Electrical-Tree Resistant Characteristics of UV-Initiated Cross-Linked Polyethylene Nanocomposites with Surface-Functionalized Nanosilica. Processes.

[28] Mojumder P., Rahman M.J., Bhuiyan M.A.H., Choudhury S. (2022). Structural and Thickness-Dependent Optical Parameters of Plasma Polymerized 2-Vinylpyridine Thin Films. Bull. Mater. Sci..

[29] Esfandyari-Manesh M., Javanbakht M., Atyabi F., Badiei A., Dinarvand R. (2011). Effect of Porogenic Solvent on the Morphology, Recognition and Release Properties of Carbamazepine-Molecularly Imprinted Polymer Nanospheres. J. Appl. Polym. Sci..

[30] Yadav S., Misra N., Khanna P., Mansi, Batra K., Khanna L. (2023). A DFT Study on Diels-Alder Reaction of Dibenzazepine and 2,5-Dimethylfuran Using Different Solvents and Temperature Conditions. Polycycl. Aromat. Compd..

[31] Rieumont J., Vega R. (1991). Studies of the Mechanism of the Radical Polymerization of Vinyl Acetate Inhibited by Furan Compounds. Phenomenology and Sensitivity Analysis. Die Makromol. Chem. Macromol. Chem. Phys..

[32] He Q., Liang J.J., Chen X., Chen S.L., Zheng H.L., Liu H.X., Zhang H.J. (2020). Removal of the Environmental Pollutant Carbamazepine Using Molecular Imprinted Adsorbents: Molecular Simulation, Adsorption Properties, and Mechanisms. Water Res..

[33] Pratama K.F., Erwanta M., Manik R., Rahayu D., Hasanah A.N. (2020). Effect of the Molecularly Imprinted Polymer Component Ratio on Analytical Performance. Chem. Pharm. Bull..

[34] Dhoot G., Auras R., Rubino M., Dolan K., Soto-Valdez H. (2009). Determination of Eugenol Diffusion through LLDPE Using FTIR-ATR Flow Cell and HPLC Techniques. Polymer.

[35] Renkecz T., László K., Horváth V. (2014). Molecularly Imprinted Microspheres Prepared by Precipitation Polymerization at High Monomer Concentrations. Mol. Imprinting.

[36] Olcer Y.A., Demirkurt M., Demir M.M., Eroglu A.E. (2017). Development of Molecularly Imprinted Polymers (MIPs) as a Solid Phase Extraction (SPE) Sorbent for the Determination of Ibuprofen in Water. RSC Adv..

[37] Meléndez-Marmolejo J., Díaz de León-Martínez L., Galván-Romero V., Villarreal-Lucio S., Ocampo-Pérez R., Medellín-Castillo N.A., Padilla-Ortega E., Rodríguez-Torres I., Flores-Ramírez R. (2022). Design and Application of Molecularly Imprinted Polymers for Adsorption and Environmental Assessment of Anti-Inflammatory Drugs in Wastewater Samples. Environ. Sci. Pollut. Res..

[38] Khasawneh O.F.S., Palaniandy P. (2021). Occurrence and removal of pharmaceuticals in wastewater treatment plants. Process Saf. Environ. Prot..

[39] Yan J., Huang J., Peng S., Sun D., Lu W., Song Z., Ma J., You J., Fan H., Chen L. (2025). Recent Advances in Molecular-Imprinting-Based Solid-Phase Microextraction for Determination of Pharmaceutical Residues. J. Chromatogr. A.

[40] Pinelli D., Foglia A., Fatone F., Papa E., Maggetti C., Bovina S., Frascari D. (2022). Ammonium Recovery from Municipal Wastewater by Ion Exchange: Development and Application of a Procedure for Sorbent Selection. J. Environ. Chem. Eng..

[41] Maggetti C., Pinelli D., Di Federico V., Sisti L., Tabanelli T., Cavani F., Frascari D. (2024). Development and validation of adsorption process for phosphate removal and recovery from municipal wastewater based on hydrotalcite–related materals. Sci. Total Environ..

