# Supplementary material for: Development and Validation of Molecularly Imprinted Polymers with Bio-Based Monomers to Adsorb Carbamazepine from Wastewater
Source: Molecules. 2025 Jun 10;30(12):2533. doi: 10.3390/molecules30122533 (PMC12196229; doi:10.3390/molecules30122533)
Supplement: Supplementary file 1 [file molecules-30-02533-s001.zip › molecules-3580052-supplementary.pdf]

Development and validation of molecularly imprinted polymers with bio-based monomers to adsorb carbamazepine from wastewater

Elettra Savigni[1], Elisa Girometti[1], Laura Sisti[1]*, Frank Bestoem[2], Davide Pinelli[1], Dario Frascari[1]
[1] Department of Civil, Chemical, Environmental and Materials Engineering, University of Bologna, Via Terracini 28, 40131 Bologna, Italy
[2] ATD GmbH, Krefelder Straße 147, D-52070 Aachen, Germany
* Correspondence: laura.sisti@unibo.it

SUPPORTING INFORMATION

Enlargement of Figure 8.

Adsorption isotherms of MIPs with bio-based monomers, conducted with actual WWTP effluent spiked with CBZ.

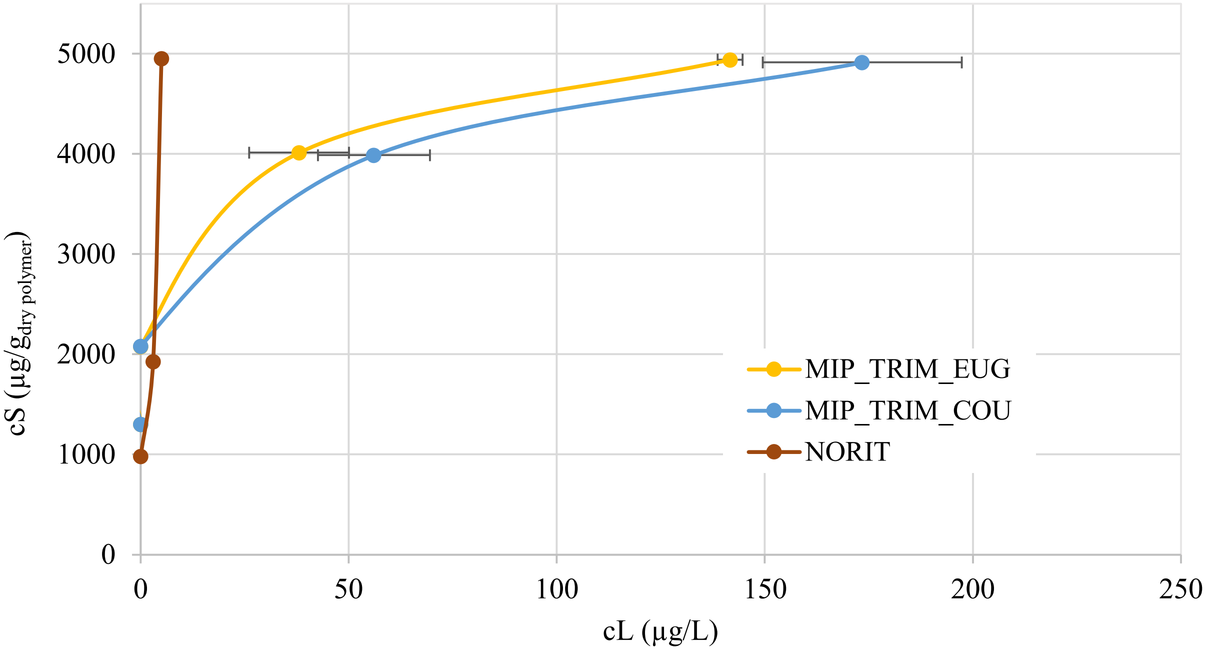

Figure S1. Adsorption isotherms of the first group of MIPs.

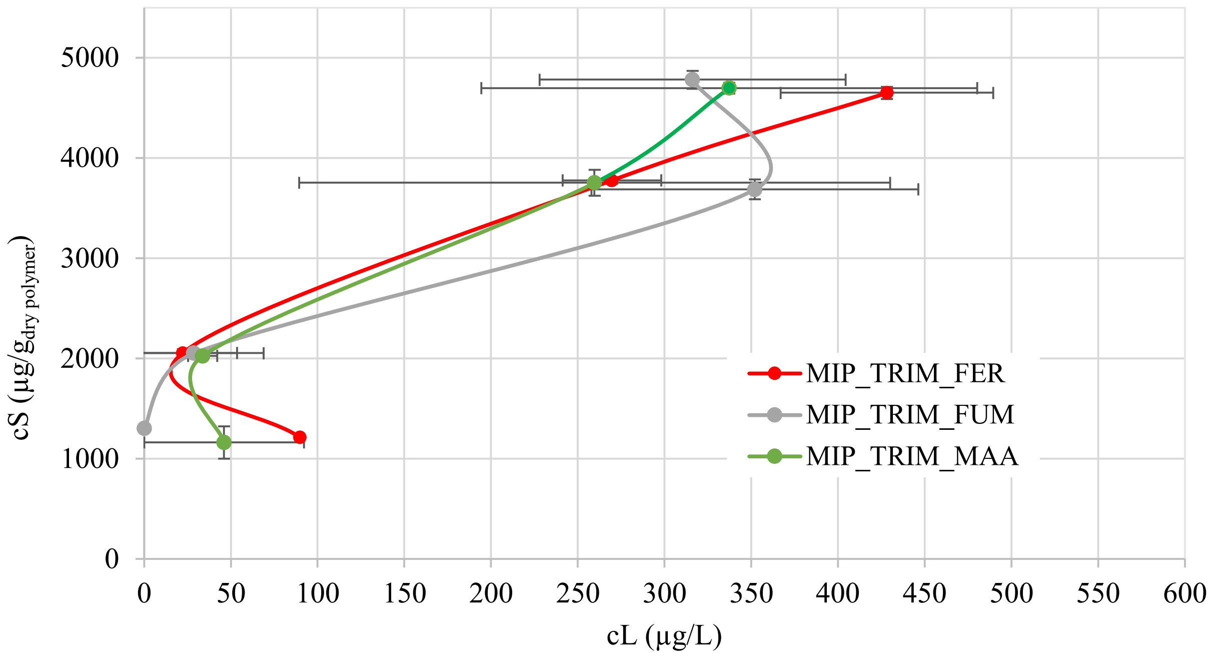

Figure S2. Adsorption isotherms of the second group of MIPs.

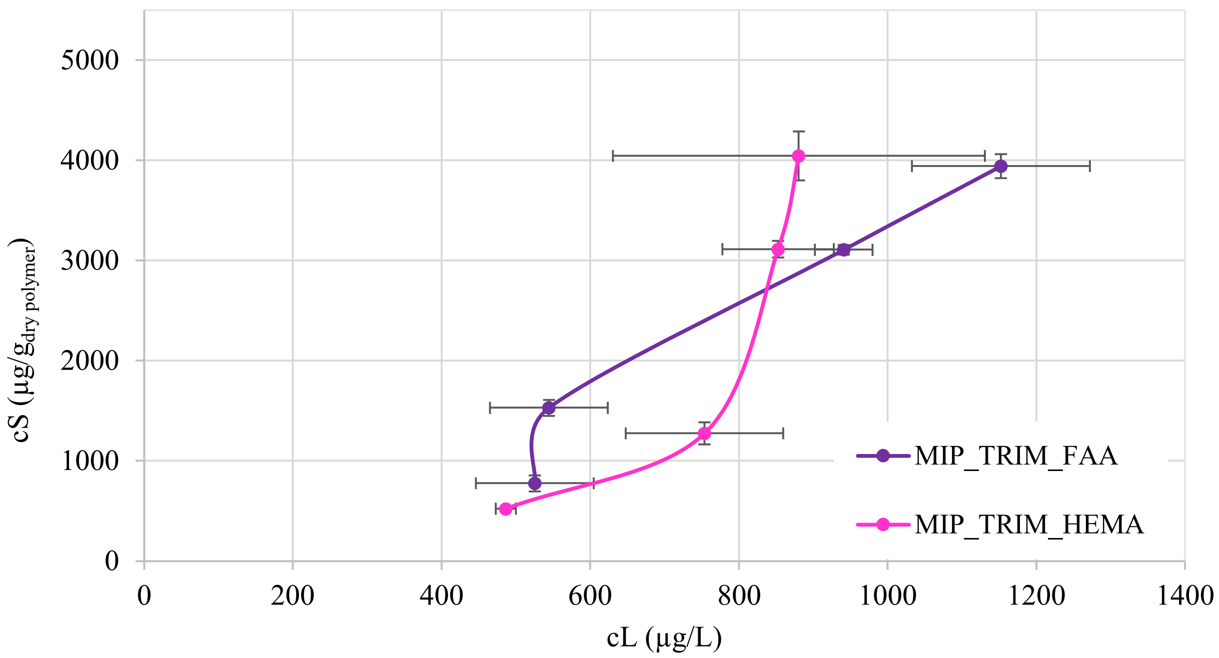

Figure S3. Adsorption isotherms of the third group of MIPs.

Text S1: Study of the interaction between CBZ and EUG

To investigate the interactions between CBZ and EUG, changes in the UV and IR spectra of CBZ-EUG mixtures were evaluated [1]. The observed modifications in the peaks are associated with the formation of a complex between the template and the monomer. Initially, CBZ was mixed with EUG (1/1 mol/mol) and analyzed by IR spectroscopy (Figures S4-S6) after 3h. The two compounds (CBZ/EUG 1/2 mol/mol) were then dissolved in an aqueous solution, and their spectra were recorded using UV spectroscopy to assess the interference of water in the complex formation. As can be seen from Figures S4-S6, the spectrum of the complex presents some perturbations if compared to the pure molecules. In particular, EUG presents OH stretching at 3515 cm^{-1} and 3445 cm^{-1} and, after interaction with CBZ, the two peaks are shifted to lower wavenumbers, 3505 cm^{-1} and 3400 cm^{-1} respectively, indicating a H-bond interaction with the amide.

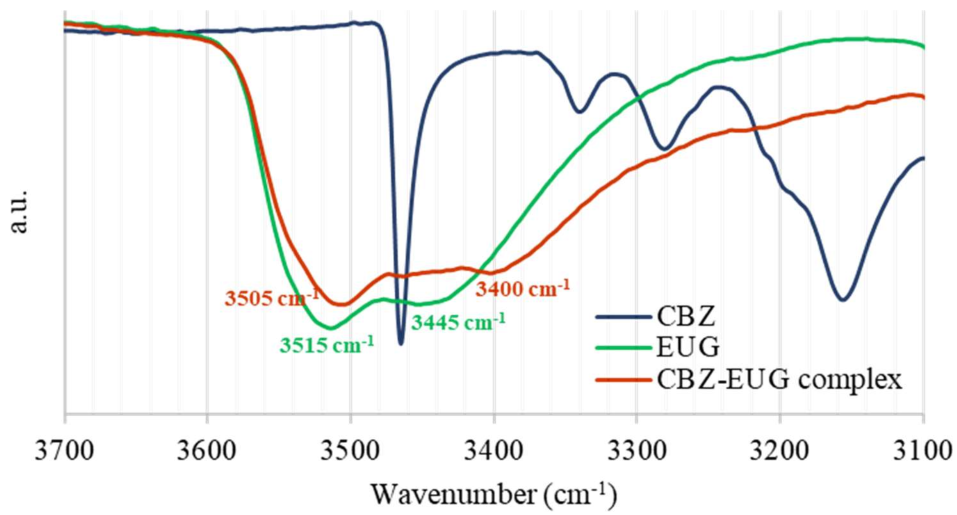

Figure S4. FT-IR spectra of CBZ and EUG mixture and pure molecules.

The presence of the complex is confirmed also by the shift of the aromatic stretching of CBZ at 1491 cm^{-1}. The light shift at higher wavenumber suggests an interaction with the aromatic ring of EUG. Other aromatic peaks of CBZ at 1605 – 1594 cm^{-1} influenced the complex spectrum, with a greater shoulder at 1603 cm^{-1}. In the complex spectrum it is also appreciable the broader peak of CBZ amide carbonyl stretching at 1665 cm^{-1}.

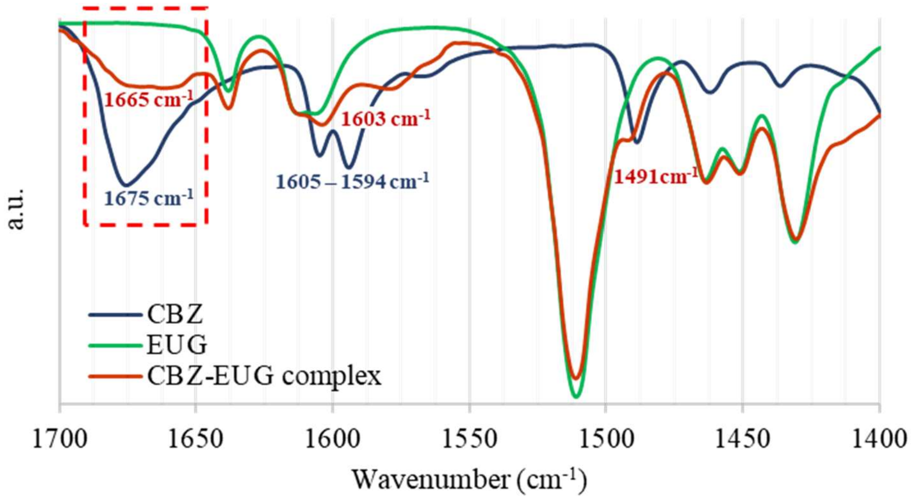

Figure S5. FT-IR spectra of CBZ and EUG mixture and pure molecules.

Finally, a slight shift in the C=C bending vibration can be observed in the fingerprint region.

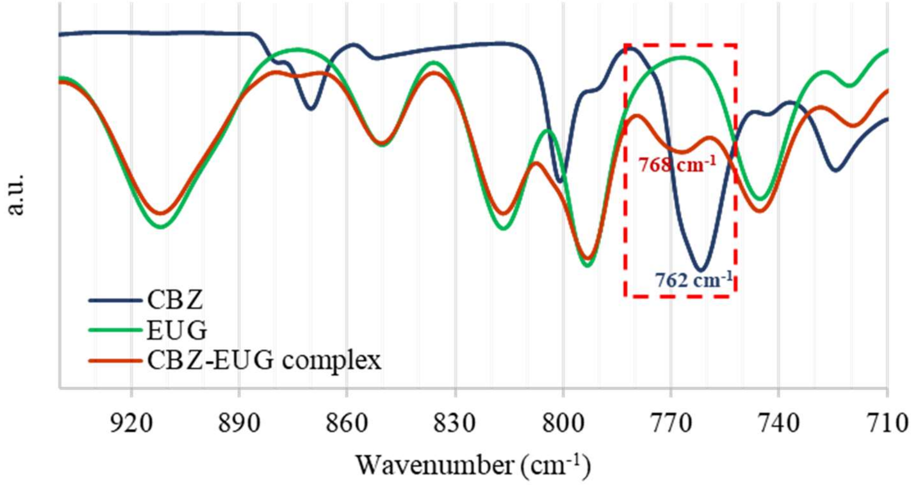

Figure S6. FT-IR spectra of CBZ and EUG mixture and pure molecules.

To investigate further the interaction between CBZ and EUG, UV spectra of water samples containing both EUG and CBZ were recorded. The solvent used has been chosen to simulate interaction in a polar environment. As shown in Figure S7, also in the water there is a shift of the peaks detected. CBZ presents two π-π* transitions at 204.5 nm due to aromatic rings and at 277.5 nm possibly related to the conjugation with carbonyl amide or the unsaturation in the central ring of carbamazepine. The hypsochromic effect detected is probably due to an interaction between the monomer and the template.

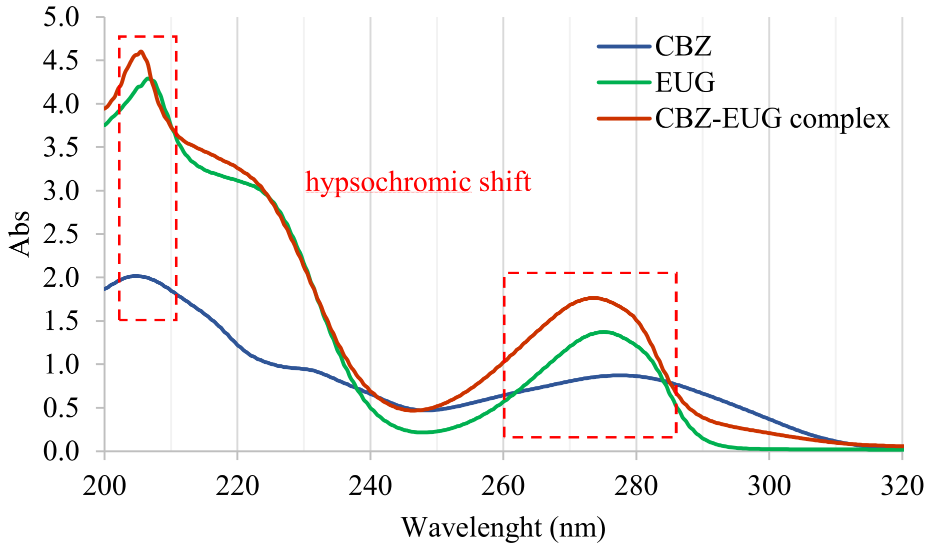

Figure S7. UV spectra of CBZ and EUG mixture and pure molecules.

Text S2: Study on kinetic adsorption

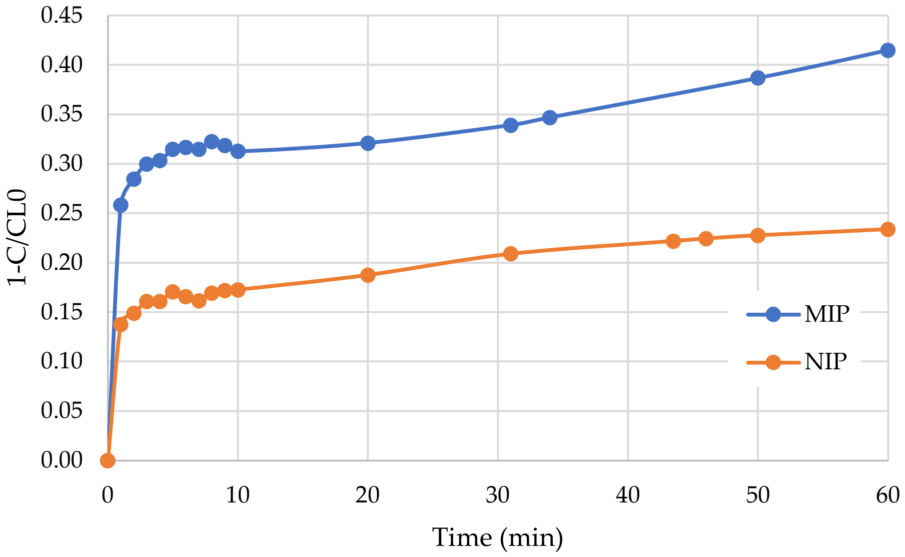

Figure S8. Kinetic adsorption study between the best performing MIP (MIP_TRIM_EUG_5-1_4h) and its corresponding NIP.

Experimental data on kinetic adsorption were interpolated by non-linear least squares using the following equations:

1. Pseudo-first-order (PFO) equation:

$$C_S = C_{S,eq}\,(1 - e^{k_1 t}) \qquad \text{(Eq. S1)}$$

where $C_{S,eq}$ (mg g^{-1}) and C_S (mg g^{-1}) are the amounts of adsorbate uptake per mass of adsorbent at equilibrium and at any time t (min), respectively; and k_1 (min^{-1}) is the rate constant of the PFO equation.

2. Pseudo-second-order (PSO) equation:

$$C_S = \frac{C_{S,eq}^2\,k_2 t}{1 + C_{S,eq} k_2 t} \qquad \text{(Eq. S2)}$$

where $C_{S,eq}$ (mg g^{-1}) and C_S (mg g^{-1}) are the amount of adsorbate adsorbed at equilibrium and at any t (min), respectively; and k_2 (g/(mg min)) is the rate constant of the PSO equation.

To calculate the kinetic parameters, nonlinear least squares were applied. Data obtained are reported in Table S1. The values show that both MIP_TRIM_EUG_5-1_4h and NIP_TRIM_EUG_5-1_4h adsorption kinetic fit better a pseudo 2nd order model, with MIP k value higher respect to NIP.

Table S1. Kinetic parameters for the best performing MIP (MIP_TRIM_EUG_5-1_4h) and its corresponding NIP.

	1st order			2nd order		
	k	$C_{s,eq}$	R2	k	$C_{s,eq}$	R2
MIP	1.292	2.450	0.881	0.880	2.565	0.922
NIP	0.771	1.586	0.754	0.566	1.736	0.853

Text S3: Study on MIP reusability

5 adsorption-desorption tests were conducted to assess MIP_TRIM_EUG_5_1_4h reusability. The desorption step was performed by feeding a solution of MeOH:Acetic acid 9:1 and samples were collected at the column outlet. The desorption curves obtained indicate that 5-10 bed volumes of regenerant were sufficient to obtain a near-complete desorption of CBZ (Figure S9).

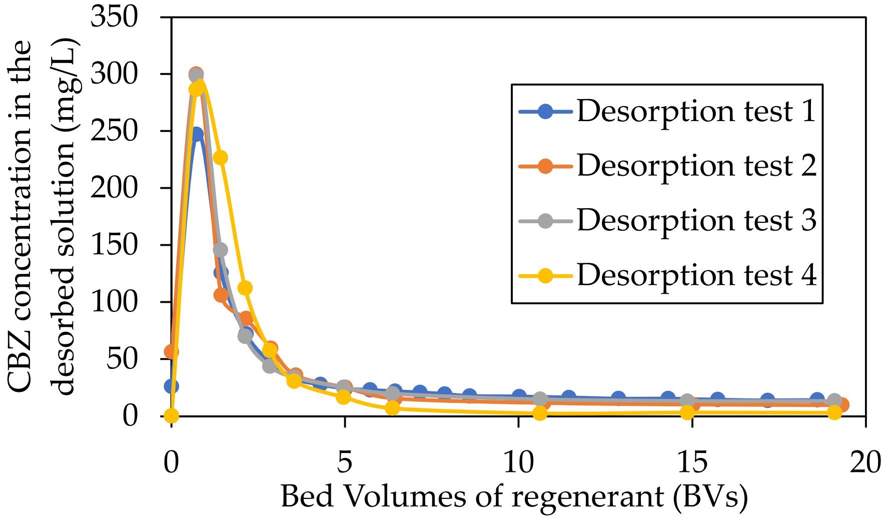

Figure S9. Desorption curves obtained in 4 repeated adsorption/desorption tests on MIP_TRIM_EUG_5_1_4h.

Text S4: Study on template removal

To verify the complete removal of CBZ from the synthesized material, the washing effluents were analyzed by HPLC to detect the amount of CBZ removed. As shown in Figure S10, the first washing step removes the majority of CBZ.

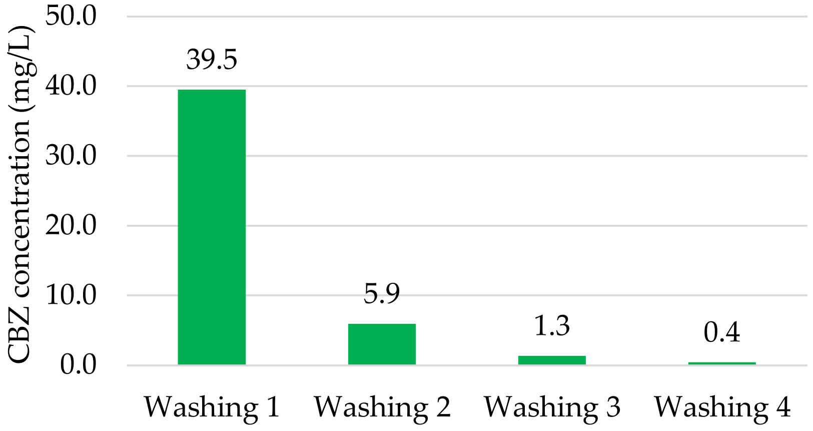

Figure S10. HPLC analysis of the washing solution for CBZ removal after polymerization.

References

[1] He, Q.; Liang, J.J.; Chen, X.; Chen, S. L.; Zheng, H. L.; Liu, H.X.; Zhang, H.J. Removal of the Environmental Pollutant Carbamazepine Using Molecular Imprinted Adsorbents: Molecular Simulation, Adsorption Properties, and Mechanisms. *Water Res*, **2020**, *168*. https://doi.org/10.1016/j.watres.2019.115164.
